# Professor Dr. Werner Bohndorf gestorben

**DOI:** 10.1007/s00066-021-01792-3

**Published:** 2021-06-08

**Authors:** Michael Flentje, Jürgen Richter

**Affiliations:** 1 Universitätsklinikum Würzburg, Klinik und Poliklinik für Strahlentherapie, Würzburg, Deutschland; 2 Würzburg, Deutschland

**Figure 1:**
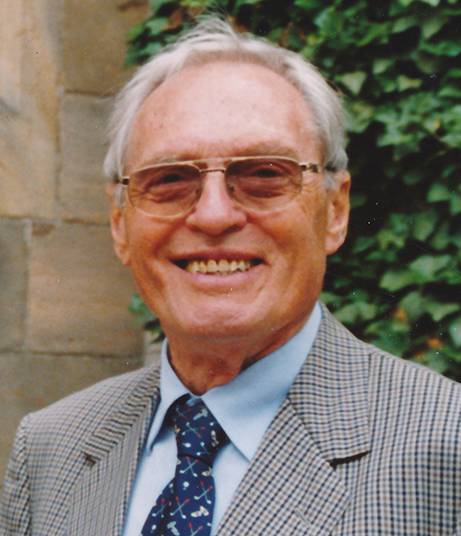

Kurz vor Vollendung seines 95. Geburtstages ist der ehemalige Direktor der Klinik und Poliklinik für Strahlentherapie der Universität Würzburg, Professor Dr. Werner Bohndorf (Abb. 1), am 9. März nach kurzer Krankheit friedlich eingeschlafen.

Werner Bohndorf wurde am 24. April 1926 in Böblingen am See nahe der Lutherstadt Eisleben geboren. Nach dem Abitur begann er 1946 mit dem Medizinstudium an der Martin-Luther-Universität in Halle und legte 1952 das Staatsexamen ab. Ab 1955 absolvierte Bohndorf die Facharztausbildung zum Radiologen an der Geschwulstklinik der Akademie der Wissenschaften in Berlin-Buch bei dem ihn prägenden Prof. Hans-Jürgen Eichhorn.

Nach der Flucht aus der DDR Anfang 1960 arbeitete er zunächst als Oberarzt im Städtischen Krankenhaus in Hanau. Im Jahr 1961 wechselte er, um wieder wissenschaftlich zu arbeiten, an die von Professor Wullstein geleitete HNO-Klinik der Würzburger Universität. Dort unterstanden ihm sowohl die Strahlentherapie wie die Röntgendiagnostik. Wissenschaftlich beschäftigte sich Bohndorf mit strahlenbiologischen Problemen und apparativen Verbesserungen für die Bestrahlungsplanung. Dies führte 1965 zur Habilitation. Im Jahr 1970 übernahm er die neu gegründete Hartstrahlenabteilung der HNO, die in die neue Kopfklinik der Würzburger Universität umzog. Mit seiner Auffassung, dass jeder Strahlentherapeut auch diagnostisch gut ausgebildet sein muss, gehörten zur Abteilung auch die entsprechenden Geräte für die Röntgendiagnostik. Die Ernennung zum Professor erfolgte 1971, und im Jahre 1974 übernahm er die Leitung der nun selbstständig gewordenen „Abteilung für Therapeutische Radiologie“. Er erhielt 1977 den Ruf auf den neuen Lehrstuhl für Strahlentherapie, dem ersten der radiologischen Fächer an der Universität Würzburg. Gleichzeitig erfolgte die Gründung der Klinik und Poliklinik für Strahlentherapie, die 1987 feierlich eingeweiht wurde. In dieser Zeit wurden zahlreiche Projekte realisiert. Dazu gehörte die Entwicklung einer Datenbank, die alle an der Klinik behandelten Patienten einschließlich der Nachsorge enthielt und dadurch umfangreiche statistische Untersuchungen ermöglichte. Die Installation eines 3D-Programms zur Berechnung von Dosisverteilungen am Universitätsrechenzentrum bildete die Voraussetzung für eine individuelle Bestrahlungsplanung und die Entwicklung neuer isozentrischer Bestrahlungstechniken, v. a. der Bewegungsbestrahlung. Bohndorf erkannte frühzeitig die große Bedeutung der Computertomographie (CT), um individuelle Körperquerschnitte für die Bestrahlungsplanung einzusetzen. Bereits zu Beginn der 1980er-Jahre wurde von der Universität ein CT angeschafft, dass zur Hälfte der Strahlentherapie zur Verfügung stand.

Mit Werner Bohndorfs ständiger Unterstützung konnten durch die Entwicklung der Computersteuerung eines Beschleunigers dynamische Bestrahlungstechniken entwickelt werden, die kommerziell erst 10 Jahre später verfügbar wurden.

Bohndorf verfasste über 120 Publikationen. Sein Auftreten in der Öffentlichkeit war zurückhaltend und bescheiden.

Nach der Emeritierung widmete er sich seinen Hobbys. Er war gemeinsam mit seiner Frau Eva-Renate ein begeisterter und erfolgreicher, Turniere gewinnender Golfspieler, der sich bis in die letzten Jahre seine körperliche Fitness erhielt. Weitere Hobbys waren das Malen, bei dem er es als Autodidakt zu hoher Fertigkeit brachte, und sein großer Garten.

Mehr als 70 Jahre stand ihm seine Frau Eva-Renate zur Seite. Die beiden Söhne folgten dem Vater und wurden Mediziner.

Prof. Bohndorfs großes Verdienst als Arzt und Wissenschaftler beruht v. a. in den Forschungen zur individualisierten Strahlentherapie sowie in deren Einführung in die klinische Routine. Viele seiner Visionen sind heute unverzichtbare Grundlage einer hochwirksamen und verträglichen Strahlenbehandlung.

Michael Flentje und Jürgen Richter

